# FOXA1-induced circOSBPL10 potentiates cervical cancer cell proliferation and migration through miR-1179/UBE2Q1 axis

**DOI:** 10.1186/s12935-020-01360-2

**Published:** 2020-08-12

**Authors:** Shanshan Yang, Yiwen Jiang, Xiaoli Ren, Dan Feng, Liaoyun Zhang, Deying He, Shiyao Hong, Li Jin, Fang Zhang, Shun Lu

**Affiliations:** 1 Department of Gynecological Radiotherapy, Harbin Medical University Cancer Hospital, No. 150 Haping Road, Nangang District, Harbin, 150081 Heilongjiang China; 2 Department of Radiotherapy, Sichuan Cancer Hospital & Institute, Sichuan Cancer Center, School of Medicine, University of Electronic Science and Technology of China, No. 55 Renmin South Road, Chengdu, 610041 Sichuan China; 3 Pharmacy Department, Sichuan Jinxin Women and Children’s Hospital, No. 66 Jingxiu Road, Jinjiang District, Chengdu, 610061 Sichuan China

**Keywords:** CircOSBPL10, miR-1179, UBE2Q1, FOXA1, CC

## Abstract

**Background:**

Recently, extensive evidence has clarified the crucial role of circular RNAs (circRNAs) as a pro-tumor or anti-cancer participant in human malignancies. A new circRNA derived from oxysterol binding protein like 10 (OSBPL10) (circOSBPL10) has not been researched in cervical cancer (CC) yet.

**Methods:**

The expression of molecules was analyzed by RT-qPCR or western blot. Several functional assays were applied to explore the biological influence of circOSBPL10 on CC. The interaction between RNAs was estimated via luciferase reporter, RNA immunoprecipitation and RNA pull-down assays.

**Results:**

CircOSBPL10 characterized with cyclic structure was revealed to possess elevated expression in CC cells. CircOSBPL10 downregulation elicited suppressive impacts on CC cell proliferation and migration. Interestingly, circOSBPL10 regulated CC progression by interacting with microRNA-1179 (miR-1179). Moreover, ubiquitin conjugating enzyme E2 Q1 (UBE2Q1) targeted by miR-1179 was positively regulated by circOSBPL10 in CC. Furthermore, enhanced UBE2Q1 expression or suppressed miR-1179 level countervailed the repressive effect of circOSBPL10 depletion on the malignant phenotypes of CC cells. Moreover, forkhead box A1 (FOXA1) was confirmed to induce circOSBPL10 expression in CC cells.

**Conclusions:**

FOXA1-induced circOSBPL10 facilitates CC progression through miR-1179/UBE2Q1 axis, highlighting a strong potential for circOSBPL10 to serve as a promising therapeutic target in CC.

## Background

As a frequent type of human gynecological malignancies worldwide, cervical cancer (CC) is depicted as one of the dominating causes contributing to cancer-associated death in women [1, 2]. It is estimated that that nearly 500,000 new cases are diagnosed with CC annually [3]. In China, CC is also regarded as one of the most prevalent lethal tumors. Over the past few years, in spite of the application of human papillomavirus (HPV) vaccine in treatment, CC is still a major stumbling block for female health [1, 4]. The majority of patients at an early stage of CC are likely to be cured through surgery [5], whereas no or limited efficient therapeutic approaches for those at the advanced stages [6]. In order to make advances in the treatment of CC, researchers have focused on exploring and developing tumor-particular biomarkers for CC [7]. For example, melatonin was identified as a new adjuvant agent in treating patients with CC [8], so was curcumin [9]. However, more efforts should be made in developing new effective therapeutic strategies for CC. Hence, it is imperative to make in-depth exploration of the underlying molecular mechanisms in CC.

Non-coding RNAs (ncRNAs) have been commonly considered as the potential key modulators in gene regulation to impact on tumor development [10, 11], including genetic and epigenetic manners [12]. In recent years, ncRNAs have been indicated as clinical biomarkers in diverse diseases [13, 14], including cancer [15, 16]. As a member of ncRNAs, circular RNAs (circRNAs) own a covalently closed structure which are tolerant to RNase R-mediated degradation [17, 18]. Increasing analyses have suggested the abnormal expression of circRNAs in various cancers [19, 20], including CC [21, 22]. Emerging researches have testified the implication of circRNAs in tumorigenesis and progression via regulation of different biological processes, which includes cell proliferation, migration and invasion [23–25]. Emerged as a new circRNA, circOSBPL10 (circbase ID: hsa_circ_0064669) is derived from back-splicing of OSBPL10 mRNA (messenger RNA). To our knowledge, the critical regulatory mechanism of circOSBPL10 has not been investigated in CC yet.

In this study, the main purpose was to decipher the potential regulatory role of circOSBPL10 in CC. Data from a series of assays uncovered that FOXA1-induced upregulation of circOSBPL10 contributes to CC progression via miR-1179/UBE2Q1 axis, revealing that circOSBPL10 might be a hopeful biomarker for CC.

## Methods

### Cell culture

Human normal cervical epithelial cells (H8) and human CC cells (C33A, CaSki, HeLa and SiHa) were bought from Chinese Academy of Sciences (Shanghai, China). Cells were cultured with Dulbecco’s Modified Eagle Medium (DMEM; Invitrogen, Carlsbad, CA, USA) adding 10% fetal bovine serum (FBS; Invitrogen), 1% penicillin/streptomycin (Sigma-Aldrich, Milan, Italy), and then incubated in an incubator of 5% CO_2_ at 37 °C.

### Cell transfection

HeLa and SiHa cells were transfected with specific short hairpin RNAs (shRNAs) against circOSBPL10 (sh-circOSBPL10#1#2), FOXA1 (sh-FOXA1#1#2) and their corresponding negative control (NC) sh-NC, as well as pcDNA3.1/circOSBPL10, pcDNA3.1/UBE2Q1, pcDNA3.1/FOXA1 and empty pcDNA3.1 (±) circRNA Mini vector, empty pcDNA3.1 vector, severally. The miR-1179 mimics, miR-1179 inhibitor, NC mimics and NC inhibitor were synthesized by GenePharma (Shanghai, China). Transfection experiments were executed by Lipofectamine 2000 (Invitrogen).

### Real-time quantitative polymerase chain reaction (RT-qPCR)

Total RNA of cells was isolated using TRIzol reagent, followed by cDNA (complementary DNA) synthesis with Reverse Transcription Kit (Invitrogen). RT-qPCR was measured by SYBR-Green Real-Time PCR Kit (Takara, Tokyo, Japan) operated on Bio-Rad CFX96 system (Takara). Relative expression level was calculated utilizing 2^−ΔΔCt^ method with normalization to glyceraldehyde-3-phosphate dehydrogenase (GAPDH) or U6. The sequences of primers were presented in Additional file 1: Table S1.

### Cell counting kit-8 (CCK-8)

In short, 1 × 10^3^ cells were plated into a 96-well plate. After incubation for specific times (24, 48, 72, 96 h), cells were processed with 10 μL of CCK-8 reagent for additional 4 h. Absorbance at 450 nm was measured via a microplate reader (Olympus, Tokyo, Japan).

### Colony formation assay

Transfected cells (1 × 10^3^) were first coated into 6-well plates. After 2 weeks of incubation, cells were rinsed with phosphate buffer saline (PBS; Sigma-Aldrich, San Francisco, USA), fixed in methanol (Sigma-Aldrich) and dyed using crystal violet (Sigma-Aldrich). The visible colonies were counted manually.

### ActinomycinD (ActD) and RNase R treatment

To block transcription, 2 mg/ml Actinomycin D (ActD; Sigma-Aldrich) or dimethylsulphoxide (DMSO; Sigma-Aldrich) was added into culture medium. Total RNA was cultivated with or without 3 U/μg of RNase R (Epicentre Technologies, Madison, WI, USA) for 30 min. After treatment with ActD or RNase R, RT-qPCR was applied for determining the expression levels of circOSBPL10 and OSBPL10 mRNA.

### Nucleic acid electrophoresis

Convergent primers and divergent primers were designed to amplify OSBPL10 mRNA or circOSBPL10. The level of circOSBPL10 in PCR products from cDNA and genomic DNA was examined by agarose gels with TE (Tris-ethylene diamine tetraacetic acid) buffer from Thermo Scientific (Waltham, MA, USA).

### Terminal deoxynucleotidyl transferase-mediated dUTP nick-end labeling (TUNEL) assay

Apoptosis transfected SiHa and HeLa cells were assessed utilizing TUNEL Apoptosis Kit (Invitrogen). 4′,6-diamidino-2-phenylindole (DAPI; Sigma-Aldrich) was employed to dye above cells. The percentage of positive stained cells was observed by fluorescence microscopy (Olympus) and then analyzed.

### Flow cytometry analysis

Cell apoptosis analysis was performed via Cell Apoptosis Analysis Kit (Takara). After incubation in 6-well plates, SiHa and HeLa cells were rinsed with PBS and resuspended in binding buffer. Followed by fixation with 70% ice-cold ethanol (Sigma-Aldrich), cells were double-stained by Annexin V-fluorescein isothiocyanate and propidium iodide. Last, cell apoptosis rate was detected by Flow Cytometer (Becton–Dickinson, MA, USA).

### Migration assay

Cell migration abilities were examined using transwell chambers (Corning, NY, USA). Transfected cells with serum-free medium were placed into top compartment, while medium with 10% FBS was added into the lower compartment. 48 h later, cells in the lower chamber were immobilized and dyed in methanol and crystal violet, separately. Then migratory cells were then counted in five random chosen fields under a microscope (Olympus).

### Wound healing

SiHa and HeLa cells were added in 6-well plates for cultivation. When cell confluence was 80%, scratches were produced in cell layer using sterile pipette tip. Afterward, cells were cleaned using PBS and incubated in a culture medium for 24 h. Images of migrating cells were detected at last.

### Western blot

Total protein was reaped in Radio Immunoprecipitation Assay (RIPA) lysis buffer (Beyotime, Shanghai, China). Bicinchoninic acid (BCA) kit (Beyotime) determined protein concentrations. Proteins were separated through sodium dodecyl sulfate–polyacrylamide gel electrophoresis (SDS-PAGE) and moved onto polyvinylidene fluoride (PVDF) membranes. The membranes were sealed with 5% skimmed milk and cultivated with primary antibodies against UBE2Q1 (orb77618, Biorbyt, San Francisco, California, USA) and GAPDH (ab8245, Abcam, Cambridge, UK). Secondary antibody was added for incubating for 1 h. GAPDH was an internal control. Proteins quantities were evaluated via chemiluminescence detection system.

### Luciferase reporter assay

The wild-type (WT) and mutant (Mut) binding sites of miR-1179 in circOSBPL10 or UBE2Q1 3′UTR was sub-cloned into pmirGLO dual-luciferase vector to construct circOSBPL10-WT/Mut or UBE2Q1-WT/Mut. And plasmids were co-transfected with miR-1179 mimics or NC mimics into SiHa and HeLa cells, respectively. The pGL3-OSBPL10 promoter was co-transfected with pcDNA3.1/FOXA1 or pcDNA3.1 into cells. Dual-Luciferase Reporter Assay System (Promega, USA) detected the luciferase activity.

#### Subcellular fractionation

Fractions of cytoplasmic and nuclear were separated using NE-PER™ Nuclear and Cytoplasmic Extraction Reagents (Invitrogen) and gathered by RNeasy Midi Kit (Qiagen, Hilden, Germany) to determine the cellular localization of circOSBPL10. RT-qPCR was used to examine the levels of circOSBPL10, U6 (nuclear control) and GAPDH (cytoplasmic control).

### RNA pull-down

Briefly, cell lysates were incubated with biotinylated RNA including Bio-miR-1179-WT, Bio-miR-1179-Mut and Bio-NC. Moreover, M-280 streptavidin magnetic beads (Sigma-Aldrich) were added to co-culture for 48 h. The relative enrichment of RNAs pulled down in each group were assayed by RT-qPCR.

### RNA immunoprecipitation (RIP)

RIP assays were progressed with Magna RIPTM RNA-Binding Protein Immunoprecipitation Kit (Millipore, Bedford, USA). SiHa and HeLa cells were lysed with RIP lysis buffer, followed by incubation with magnetic beads conjugated with anti-Ago2 (Millipore) or anti-IgG (Millipore). The RT-qPCR was performed to evaluate the expression levels of circOSBPL10, miR-1179 and UBE2Q1 in the precipitates.

### Chromatin immunoprecipitation (ChIP)

Via Magna ChIP Kit (Millipore), ChIP experiment was achieved. DNA in cell lysates was interrupted into 200-300-bp chromatin fragments by ultrasound. After that, lysates were subjected to immunoprecipitation with anti-FOXA1 or anti-IgG (negative control group). The precipitated DNA fragments were detected by RT-qPCR.

### Statistical analysis

Experimental data from at least three independent experiments were shown as mean ± standard deviation (SD). Statistical analysis was performed using GraphPad Prism 7.0 software (Graph Pad, La Jolla, CA, USA). Significance in differences between 2 or more groups was analyzed via student’s *t* test or one-way analysis of variance (ANOVA). P < 0.05 had statistical significance in requirements.

## Results

### CircOSBPL10 is highly expressed in CC and its depletion impedes CC cell proliferation and migration

**Fig. 1:**
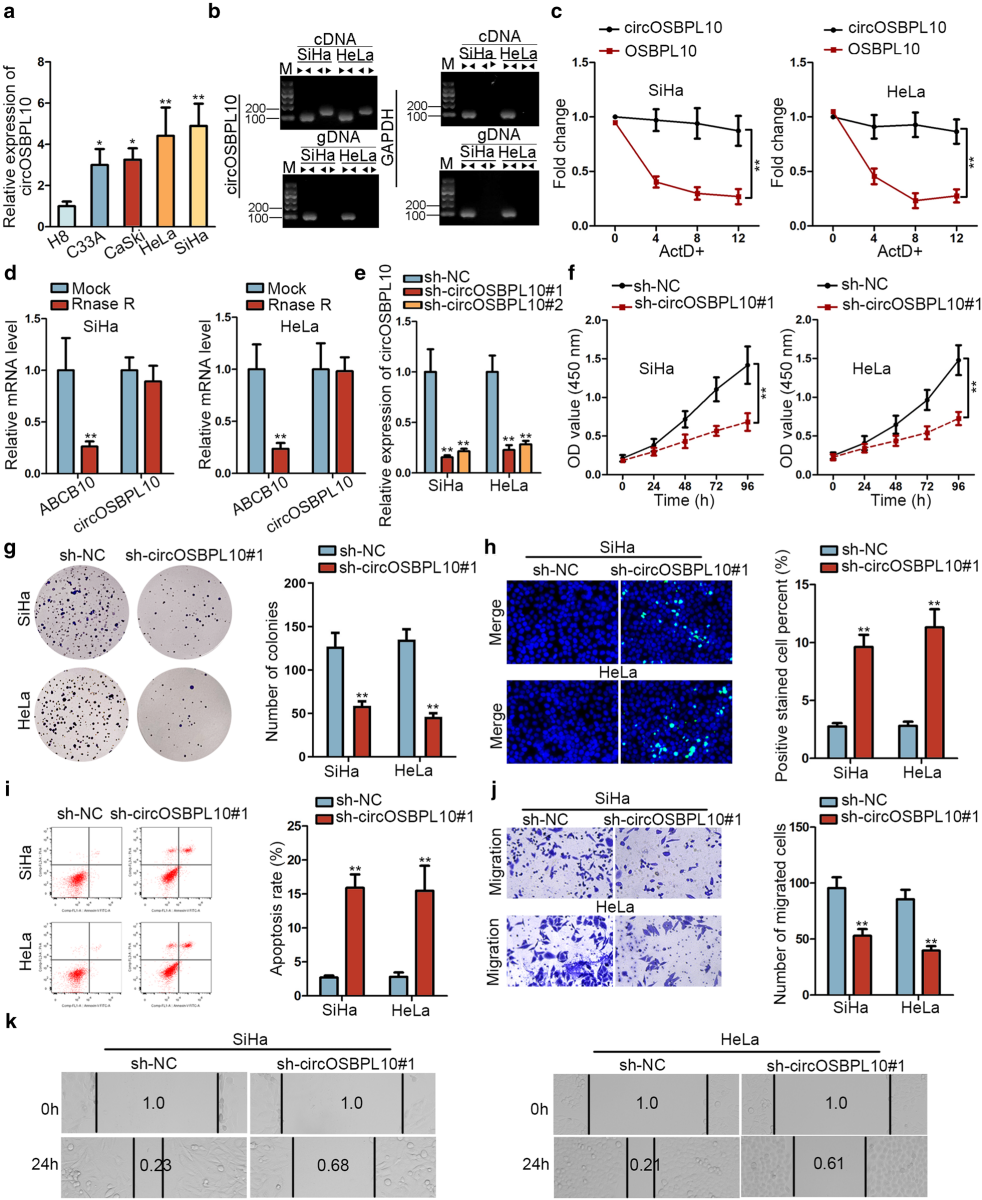
Circular RNA circOSBPL10 was highly expressed in CC and knockdown of it suppressed CC progression. **a** CircOSBPL10 expression was detected by RT-qPCR in CC cell lines H8 cells. **b** It was delineated by nucleic acid electrophoresis analysis that divergent primers amplified circOSBPL10 from cDNA, but not from gDNA. GAPDH was a negative control. **c** The resistance of circOSBPL10 and OSBPL10 mRNA to ActD in SiHa and HeLa cells was analyzed by RT-qPCR. **d** RT-qPCR assay was conducted to determine the abundance of circOSBPL10 and linear OSBPL10 mRNA in SiHa and HeLa cells treated with RNase R (normalized to mock treatment). **e** RT-qPCR was utilized to analyze the efficacy of circOSBPL10 knockdown in SiHa and HeLa cells. **f**, **g** The proliferation ability of SiHa and HeLa cells transfected with sh-circOSBPL10#1 or sh-NC was evaluated via CCK-8 and colony formation. **h, i** Cell apoptosis ability in transfected cells was measured by TUNEL assay and flow cytometry analysis. **j**, **k** Transwell and wound healing assays were conducted to analyze the migration of transfected cells. *P < 0.05, **P < 0.01

To study the cellular function of circOSBPL10 in CC, we first applied RT-qPCR analysis and unveiled a marked elevation of circOSBPL10 expression in CC cell lines compared with H8 cells (Fig. 1a). Then, nucleic acid electrophoresis manifested that in SiHa and HeLa cells, divergent primers could produce circOSBPL10 from cDNA but not from genomic DNA (gDNA), while convergent primers amplified linear OSBPL10 from both cDNA and gDNA (Fig. 1b). Besides, OSBPL10 mRNA was greatly degraded by ActD whereas circOSBPL10 exhibited as resistant to ActD (Fig. 1c). Additionally, OSBPL10 expression was dramatically reduced whereas circOSBPL10 expression demonstrated no evident change after SiHa and HeLa cells were treated with RNase R (Fig. 1d). Then, we verified that circOSBPL10 expression was lowered in two CC cells after transfection with sh-circOSBPL10#1/2, while those with sh-circOSBPL10#1 showed higher silencing efficiency (Fig. 1e). Subsequently, cell proliferation assays depicted a notably weakened proliferation ability of SiHa and HeLa cells under circOSBPL10 silence (Fig. 1f, g). Moreover, cell apoptosis capability was proved to be facilitated after silencing circOSBPL10 in SiHa and HeLa cells (Fig. 1h, i). Furthermore, it was uncovered that circOSBPL10 deficiency gave rise to attenuated migration ability of SiHa and HeLa cells (Fig. 1j, k). Taken together, circOSBPL10 is expressed at high levels in CC and knockdown of it impairs malignant behaviors in CC cells.

### CircOSBPL10 sponges miR-1179 in CC

**Fig. 2:**
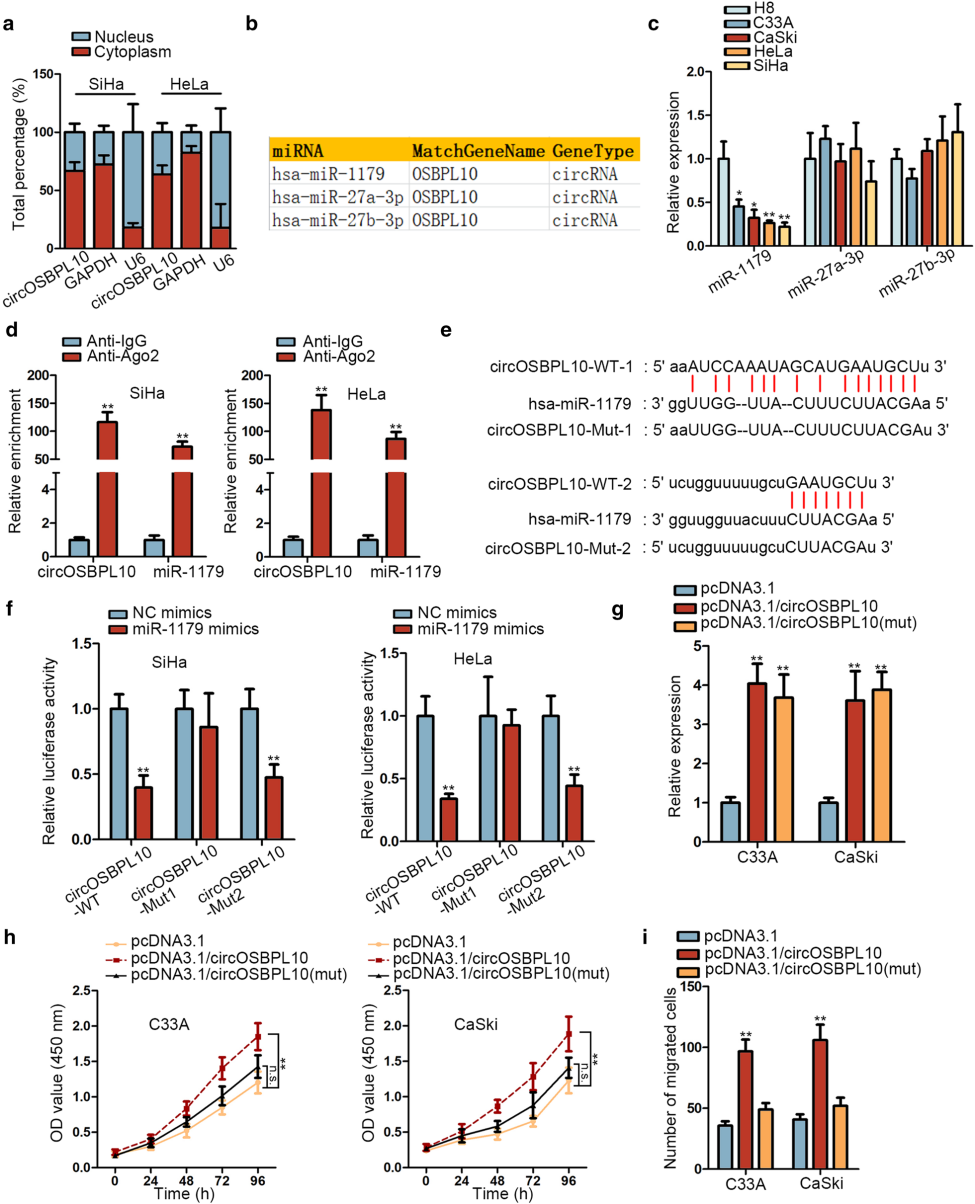
CircOSBPL10 sponged miR-1179 in CC. **a** Subcellular fractionation was applied to detect the cellular sublocalization of circOSBPL10 in SiHa and HeLa cells. **b** MiR-1179, miR-27a-3p and miR-27b-3p were predicted via starBase to have the binding capacity with circOSBPL10. **c** MiR-1179 expression in CC cell lines and H8 cells was detected via RT-qPCR. **d** RIP assay unveiled the significant enrichment of circOSBPL10 and miR-1179 in anti-Ago2 group. **e** Two binding sites between circOSBPL10 and miR-1179 were predicted via starBase. **f** Luciferase reporter assay validated the interaction between circOSBPL10 and miR-1179. **g** The expression of circOSBPL10 in SiHa and HeLa cells transfected with pcDNA3.1, pcDNA3.1/circOSBPL10 or pcDNA3.1/circOSBPL10 (mut) was analyzed through RT-qPCR. **h, i** Cell proliferation and migration in different groups was analyzed via CCK-8 and transwell assays, respectively. *P < 0.05, **P < 0.01

For the purpose of investigating the molecular mechanism of circOSBPL10 in regulating CC, we first detected its cellular sublocalization in SiHa and HeLa cells via subcellular fractionation. As illustrated in Fig. 2a, circOSBPL10 was majorly distributed in cytoplasm. Thus, we speculated that circOSBPL10 might affect CC via serving as a sponge of specific miRNA. After searching starBase (http://starbase.sysu.edu.cn/) with certain condition (CLIP Data: strict stringency ≥ 5, Degradome Data: low stringency ≥ 1), three miRNAs (miR-1179, miR-27a-3p and miR-27b-3p) were revealed to have binding potentials with circOSBPL10 (Fig. 2b). Then, we discovered a significant downregulation of miR-1179, whereas no apparent changes on the levels of miR-27a-3p and miR-27b-3p, in CC cell lines compared to normal H8 cells (Fig. 2c). Therefore, miR-1179 was chosen for further analysis. Subsequently, circOSBPL10 and miR-1179 were presented to be conspicuously concentrated in anti-Ago2 group (Fig. 2d). Afterwards, two binding sites between circOSBPL10 and miR-1179 were predicted via starBase (Fig. 2e). Moreover, we validated that miR-1179 bound with circOSBPL10 at site 1 (Fig. 2f). To further test whether circOSBPL10 promoted CC progression via its interaction with miR-1179, we mutated the sequence of circOSBPL10 recognized by miR-1179. As displayed in Fig. 2g, the expression of circOSBPL10 was observably elevated in C33A and CaSki cells after overexpressing circOSBPL10 and circOSBPL10 (mut). Interestingly, it seemed that cell proliferation and migration in C33A and CaSki cells could be fortified by overexpression of full-length circOSBPL10 but not by upregulation of circOSBPL10 with mutated miR-1179 binding sites (Fig. 2h, i), indicating that the function of circOSBPL10 in CC depended on its binding to miR-1179. In conclusion, circOSBPL10 interacts with miR-1179 to drive CC progression.

### CircOSBPL10 upregulates UBE2Q1 level in CC by sequestering miR-1179

**Fig. 3:**
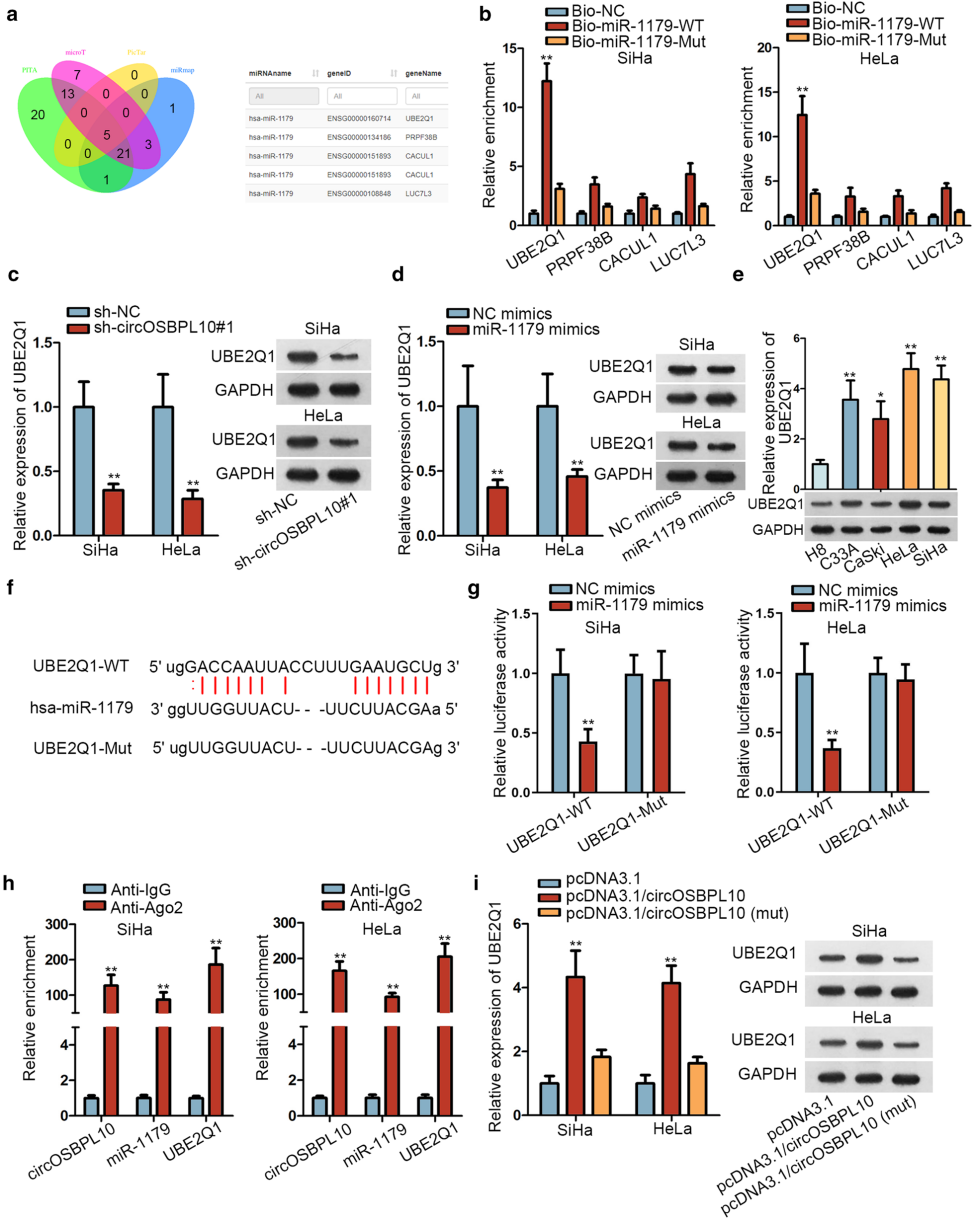
CircOSBPL10 upregulated UBE2Q1 expression by competitively binding with miR-1179 in CC. **a** Venn diagram showed the overlaps of potential miR-1179 targets predicted by PITA, microT, PicTar, miRmap. **b** The binding capacity between miR-1179 and four mRNAs was verified through RNA pull-down assay. **c**, **d** The expression of UBE2Q1 in SiHa and HeLa cells transfected with different plasmids was detected via RT-qPCR and western blot. **e** UBE2Q1 expression in CC cell lines and H8 cells was detected via RT-qPCR. **f** The binding sites between UBE2Q1 and miR-1179 obtained from starBase were displayed. **g** The interaction between UBE2Q1 and miR-1179 was validated by luciferase reporter assay. **h** RIP analysis revealed that circOSBPL10, miR-1179 and UBE2Q1 co-existed in RISCs (RNA-induced silencing complexes). **i** RT-qPCR and western blot were utilized to detect the expression of UBE2Q1 in indicated cells. **P < 0.01

To further investigate the downstream mechanism of circOSBPL10 in CC, starBase was utilized. As predicted under certain circumstances (CLIP Data: strict stringency ≥ 3, Degradome Data: low stringency ≥ 1), four candidates (UBE2Q1, PRPF38B, CACUL1 and LUC7L3) were found as the targets of miR-1179 (Fig. 3a). The following RNA pull-down assay demonstrated the distinct enrichment of UBE2Q1 whereas limited harvest of other three mRNAs in Bio-miR-1179-WT group (Fig. 3b). More importantly, UBE2Q1 level was cut down in SiHa and HeLa cells by circOSBPL10 knockdown as well as by augmented miR-1179 expression (Fig. 3c, and Additional file 2). Besides, RT-qPCR obtained a conspicuous elevation on UBE2Q1 expression in CC cell lines relative to H8 cells, at both mRNA and protein levels (Fig. 3e and Additional file 2). Afterwards, the binding sites between UBE2Q1 and miR-1179 were obtained through starBase prediction (Fig. 3f). Importantly, we observed declined luciferase activity of UBE2Q1-WT owing to miR-1179 upregulation through luciferase reporter assay. However, no overt changes on that of UBE2Q1-Mut were noted between two groups (Fig. 3g). More importantly, a significant enrichment of circOSBPL10, miR-1179 and UBE2Q1 in Anti-Ago2 groups was observed via RIP analysis (Fig. 3h). Further, UBE2Q1 expression was visibly upregulated by circOSBPL10 overexpression, whereas enhanced expression of mutated circOSBPL10 had no such influence on UBE2Q1 expression in these two CC cells (Fig. 3i and Additional file 2). In sum, circOSBPL10 facilitates UBE2Q1 expression by sponging miR-1179 in CC.

### CircOSBPL10 accelerates CC progression via miR-1179/UBE2Q1 axis

**Fig. 4:**
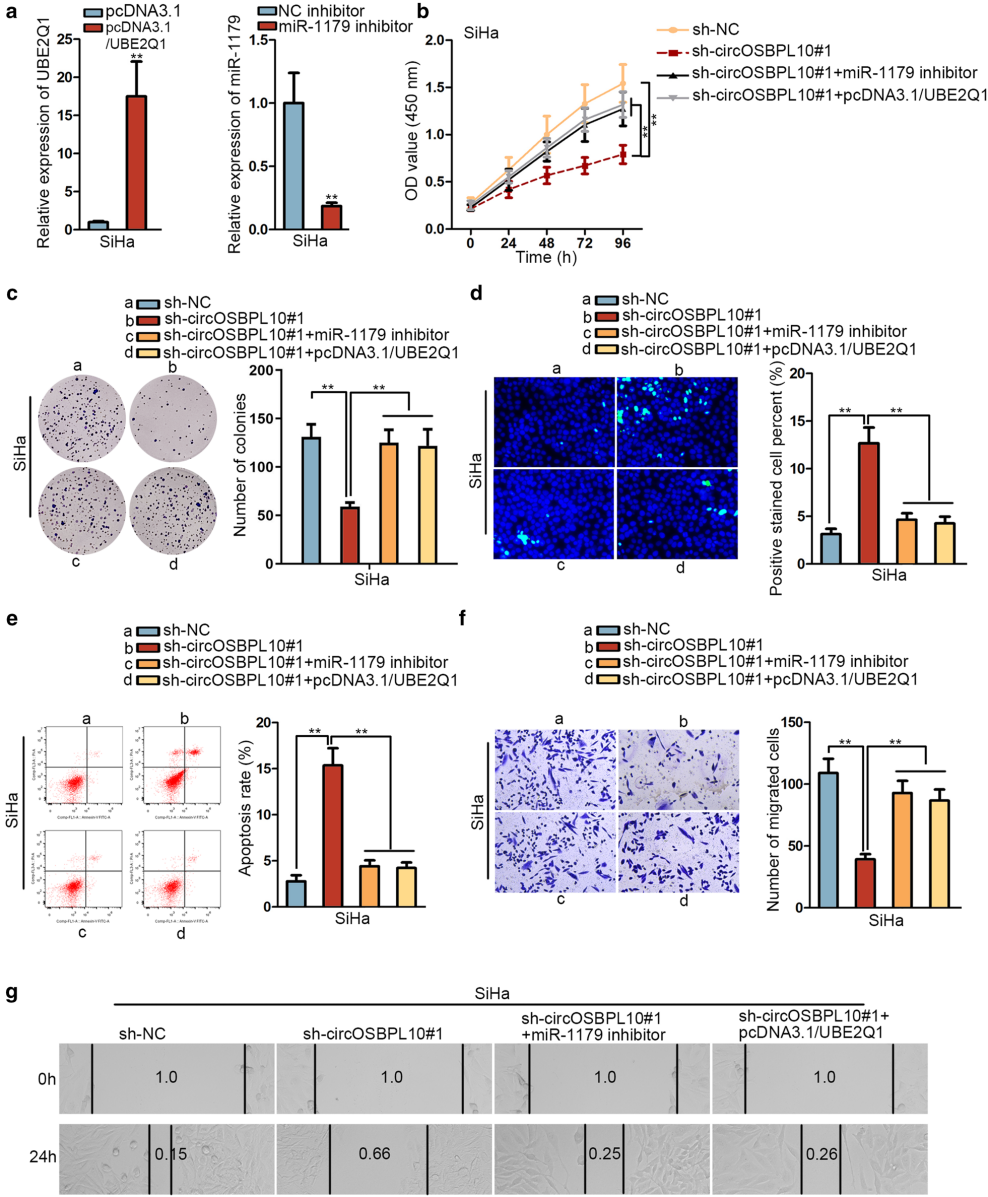
CircOSBPL10 accelerated CC progression via miR-1179/UBE2Q1 axis. **a** The efficacy of UBE2Q1 overexpression and miR-1179 inhibition in SiHa cells was analyzed by RT-qPCR. **b, c** The proliferation ability of SiHa cells under diverse conditions were measured by CCK-8 and colony formation assays. **d, e** The apoptosis ability of transfected cells were measured via TUNEL assay and flow cytometry analysis. **f**, **g** Transwell and wound healing assays were carried out to analyze cell migration under different contexts. **P < 0.01

Given that circOSBPL10 sponged miR-1179 to upregulate UBE2Q1 expression in CC, we intended to detect whether this mechanism contributed to CC progression. Herein, the efficacy of elevating UBE2Q1 expression or lowering miR-1179 level was analyzed at first by RT-qPCR and the outcome appeared to be satisfactory in SiHa cells (Fig. 4a). Afterwards, we testified that UBE2Q1 upregulation or miR-1179 inhibition could offset circOSBPL10 depletion-mediated suppressive effect on cell proliferation (Fig. 4b, c). In addition, cell apoptosis facilitated by circOSBPL10 knockdown was counteracted by UBE2Q1 overexpression or miR-1179 suppression (Fig. 4d, e). Moreover, the attenuated cell migration capability in circOSBPL10-depleted cells was restored by upregulation of UBE2Q1 or inhibition of miR-1179 (Fig. 4f, g). To conclude, circOSBPL10 promotes CC progression via miR-1179/UBE2Q1 axis.

### FOXA1 activates circOSBPL10 expression in CC

**Fig. 5:**
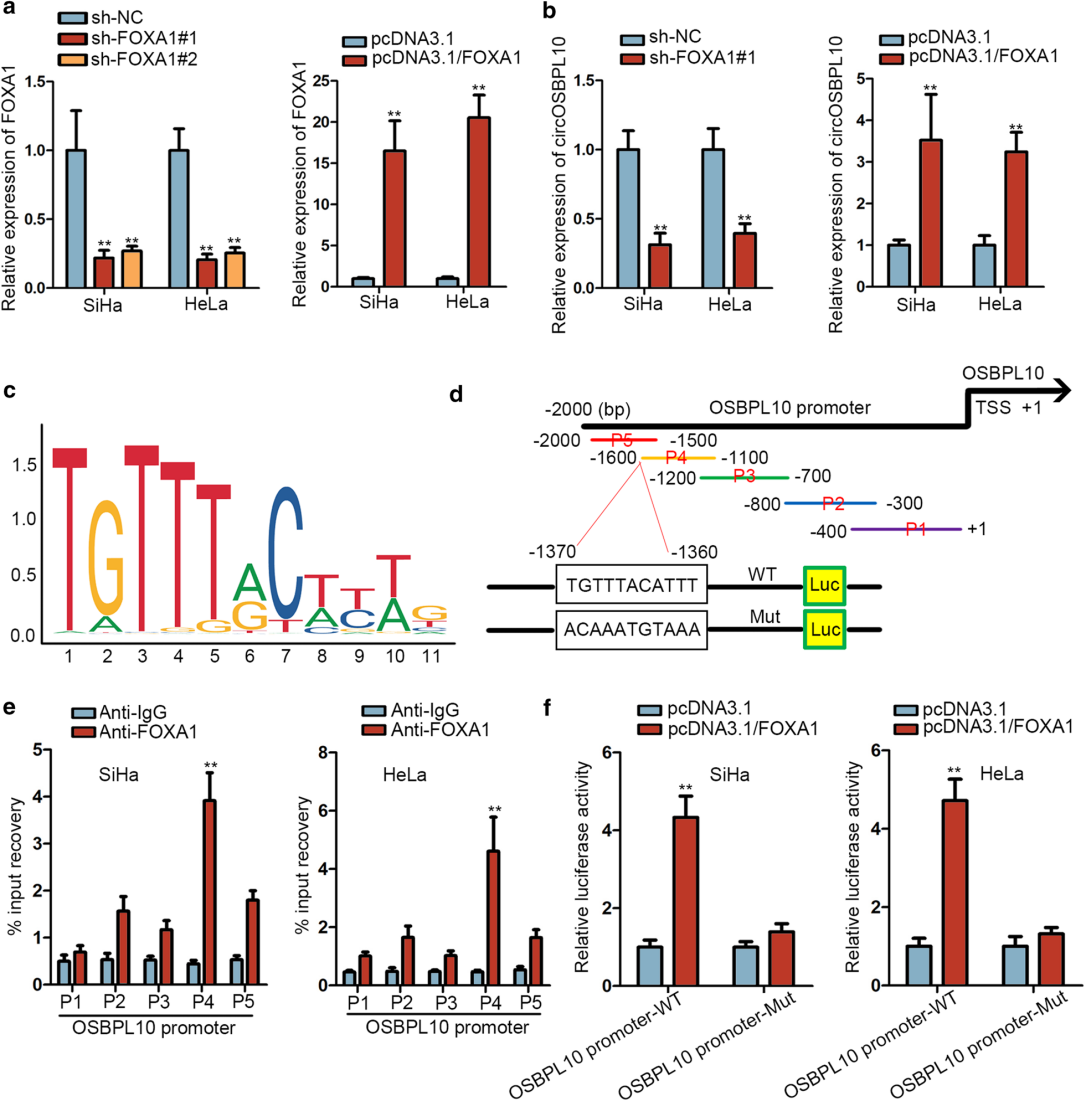
FOXA1 activated circOSBPL10 expression in CC. **a** The efficacy of FOXA1 knockdown and overexpression in SiHa and HeLa cells was obtained through RT-qPCR analysis. **b** The expression of circOSBPL10 in different groups was detected by RT-qPCR. **c** The DNA motif of FOXA1 was obtained from JASPAR database. **d** Full-length of OSBPL10 promoter was fragmented into 5 pieces (P1-5), and JASPAR predicted a potential binding site for FOXA1 to OSBPL10 promoter at P4 region. **e** ChIP assay proved that the P4 region of OSBPL10 promoter was recognized by FOXA1 in both SiHa and HeLa cells. **f** The interaction between OSBPL10 promoter and FOXA1 at above predicted sites was testified by luciferase reporter assay. **P < 0.01

Since the downstream signaling responsible for the regulation of circOSBPL10 in CC had been studied, we subsequently focused on figuring out its possible upstream mechanism. Through utilizing UCSC (University of California, Santa Cruz: http://genome.ucsc.edu/), FOXA1 seemed to be a probable transcription factor of OSBPL10 (the host gene of circOSBPL10). Prior to testify the influence of FOXA1 on circOSBPL10 expression in CC, we first silenced or overexpressed FOXA1 with satisfactory efficacies in SiHa and HeLa cells (Fig. 5a). Of interest, we discovered that the expression of circOSBPL10 was distinctly declined by FOXA1 depletion whereas overtly augmented by FOXA1 upregulation (Fig. 5b). Later on, we employed JASPAR database (http://jaspar.genereg.net/) and obtained the DNA motif of FOXA1 (Fig. 5c). Seen from Fig. 5d, the sequences of OSBPL10 promoter were fragmented into five parts (P1-5). Interestingly, it was verified by ChIP assay that FOXA1 mainly bound to OSBPL10 promoter at P4 region (Fig. 5e). Moreover, when overexpressing FOXA1 in SiHa and HeLa cells, the luciferase activity of OSBPL10 promoter-WT was observably increased while there were no evident changes on that of OSBPL10 promoter-Mut (with mutated FOXA1 binding sites predicted in P4 region) (Fig. 5f). In a word, FOXA1 activates OSBPL10 transcription and thereby facilitates circOSBPL10 expression in CC.

## Discussion

Mounting evidence has manifested that circRNAs serve vital parts in CC initiation and progression. For instance, circRNA SMARCA5 regulates CC progression by sponging miR-620 [26]. Increased expression of circ_0067934 accelerates CC development by targeting miR-545/EIF3C axis [27]. CircRNA hsa_circ_0023404 plays an oncogenic part in CC [28]. CircOSBPL10, derived from back-splicing of OSBPL10 mRNA, is a circular RNA that has not been studied in CC but is worth exploring. In this research, circOSBPL10 was evidenced to have a circular structure and possess high levels in CC. Additionally, silenced circOSBPL10 exerted suppressive impacts on CC cell proliferation and migration.

Increasing researches have elucidated that circRNAs might serve as a molecular sponge of specific miRNAs, which has been suggested to be of significant value in CC [29, 30], so as to regulate the tumorigenesis and development of numerous cancers including CC [22, 23]. In current study, on the basis of bioinformatics prediction and molecular mechanism experiments, miR-1179 that was unveiled as an anti-tumor regulator in some cancers [31, 32], was screened out and validated to be implicated in circOSBPL10- regulated cellular processes in CC.

Previously, UBE2Q1 has been depicted as a critical participator in tumor progression [33, 34]. In present study, UBE2Q1 was verified capable of binding with miR-1179, and its expression was boosted by circOSBPL10 in CC through miR-1179 sequestration. More importantly, the follow-up rescue experiments indicated that UBE2Q1 upregulation or miR-1179 inhibition could rescue circOSBPL10 depletion-mediated suppressive effects on malignant phenotypes in CC.

Increasing researches have indicated that FOXA1 expresses at high levels in many cancers, such as lung cancer [35], glioma [36] and prostate cancer [37]. Besides, a previous study indicated that FOXA1 could directly bind with PLOD2 promoter and activate PLOD2 transcription in NSCLC [38]. Similarly, we revealed that FOXA1 activates OSBPL10 transcription and thereby facilitates circOSBPL10 expression in CC in this study.

## Conclusion

In conclusion, FOXA1-induced circOSBPL10 promotes CC progression by targeting miR-1179/UBE2Q1 axis, providing novel insights into exploring more effective treatment of CC. However, the biggest regret is that no clinical data are included in this work, and the findings in our study need to be further testified by clinical samples in the future.

## Supplementary information

**Additional file 1: Table S1.** List of the sequences of primers used in RT-qPCR.

**Additional file 2: Figure S1.** The size of marker for western blot gels.

## Data Availability

Research data and material are not shared.

## Abbreviations

circRNA: Circular RNA
CC: Cervical cancer
UBE2Q1: Ubiquitin conjugating enzyme E2 Q1
FOXA1: Forkhead box A1
ncRNAs: Noncoding RNAs
FBS: Fetal bovine serum
ActD: Actinomycin D
gDNA: Genomic DNA
