# Supplementary figures and images for: FOXA1-induced circOSBPL10 potentiates cervical cancer cell proliferation and migration through miR-1179/UBE2Q1 axis
Source: Cancer Cell Int. 2020 Aug 12;20:389. doi: 10.1186/s12935-020-01360-2 (PMC7422615; doi:10.1186/s12935-020-01360-2)

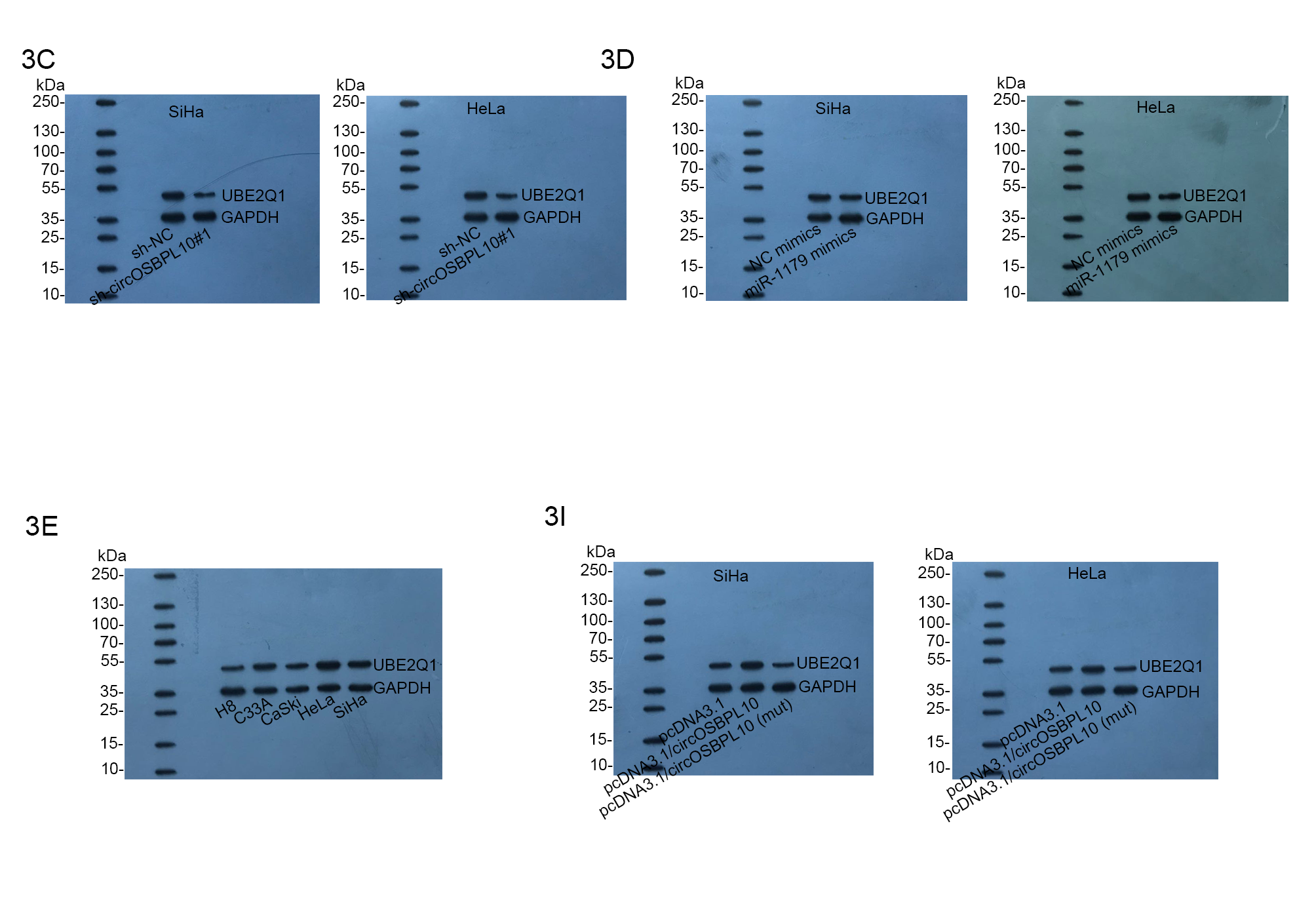

Supplement: Supplementary file 2 — Additional file 2: Figure S1. The size of marker for western blot gels. [file 12935_2020_1360_MOESM2_ESM.tiff]
